# Efficacy of fluocinolone acetonide implant in diabetic macular edema patients previously treated with dexamethasone implant: A systematic review and meta-analysis

**DOI:** 10.12669/pjms.41.8.12675

**Published:** 2025-08

**Authors:** Weiping Hu, Liyang Ni, Huangfang Ying, Zilong Zhang, Shudan Tu, Yonggang Tao, Lanping Chen

**Affiliations:** 1 Weiping Hu Department of Ophthalmology, Affiliated Hospital of Shaoxing University, Shaoxing, Zhejiang Province 312000, P.R. China; 2 Liyang Ni Department of Ophthalmology, Affiliated Hospital of Shaoxing University, Shaoxing, Zhejiang Province 312000, P.R. China; 3 Huangfang Ying Department of Ophthalmology, Affiliated Hospital of Shaoxing University, Shaoxing, Zhejiang Province 312000, P.R. China; 4 Zilong Zhang Department of Ophthalmology, Affiliated Hospital of Shaoxing University, Shaoxing, Zhejiang Province 312000, P.R. China; 5 Shudan Tu Department of Ophthalmology, Affiliated Hospital of Shaoxing University, Shaoxing, Zhejiang Province 312000, P.R. China; 6 Yonggang Tao Department of Ophthalmology, Affiliated Hospital of Shaoxing University, Shaoxing, Zhejiang Province 312000, P.R. China; 7 Lanping Chen Department of Anesthesia Operation, Affiliated Hospital of Shaoxing University, Shaoxing, Zhejiang Province 312000, P.R. China

**Keywords:** Diabetic retinopathy, Fluocinolone acetonide, Macular edema, Steroids

## Abstract

**Background & Objective:**

Several diabetic macular edema (DME) patients are resistant to anti-vascular endothelial growth factors (VEGF) injections and are treated with corticosteroid implants. Dexamethasone implants (DEXI) are popular, but fluocinolone acetonide implants (FACI) are being used as third-line DME therapy, given their convenience and prolonged action. The efficacy of FACI in patients previously treated with DEXI remains understudied. We collated evidence from single-arm studies examining the efficacy and safety of FACI in DME patients treated with DEXI.

**Methodology:**

We explored the websites of Embase, PubMed, PubMed CENTRAL, Web of Science, and Scopus up to January 15, 2025. Pre and post-FACI best-corrected visual acuity (BCVA), central retinal thickness (CRT), and intraocular pressure (IOP) were compared.

**Results:**

Eight studies with 365 eyes were included. Meta-analysis showed that use of FACI was associated with statistically significant improvement in BCVA (letters) at three months (MD: 5.40 95% CI: 0.13, 10.67 I^2^= 78%) but not at six months (MD: 5.43 95% CI: 1.79, 12.64 I^2^=86%), and 12 months (MD: 3.39 95% CI: 3.12, 9.91 I^>2^=84%). The pooled analysis indicated a statistically significant reduction in CRT after administration of FACI at 3 months (MD: 118.11 95% CI: 211.62, -24.61 I^2^=93%), six months (MD: 121.79 95% CI: 212.35, -31.24 I^2^=94%), and 12 months (MD: 108.33 95% CI: 175.47, -41.2 I^2^=92%). No significant change in IOP was noted at all follow-up intervals.

**Conclusions:**

Use of FACI in patients previously treated with DEXI results in anatomical improvement but may be associated with limited visual gain. The use of FACI seems safe without significant change in IOP.

***Registration:*** PROSPERO CRD42025635691.

## INTRODUCTION

The prevalence of diabetes mellitus is expected to increase exponentially by 2050.^1^ This would also correspondingly increase the prevalence of diabetic macular edema (DME) which is the fourth leading cause of visual impairment worldwide.^2^ DME is seen in roughly 8% of diabetic patients in the USA alone leading to heavy medico-economic impact due to the burden of care, particularly in the working class.^3^

The pathophysiology of DME is complex and several inflammatory and vascular mechanisms have been implicated. Hyperglycemia of diabetes is associated with increased vascular endothelial factor (VEGF) levels which increases permeability and damages the retinal capillaries. Leakage of serous blood components causes fluid accumulation in the intra and subretinal regions leading to macular thickening.^4^,^5^ Drugs targeting the pathway, i.e., the anti-VEGF class namely bevacizumab, ranibizumab, and aflibercept have therefore become the first line of therapy in the treatment of DME.^6^ However, drug resistance and relapse are common problems in as many as 40% of cases treated with anti-VEGF.^7^ This gap is filled with intravitreal steroids which diminish the production of VEGF and inflammatory mediators and have become the second-line of therapy for DME. The sustained-release intravitreal 0.7 mg dexamethasone implant (DEXI), Ozurdex® (AbbVie Inc., North Chicago, IL, USA) was the first corticosteroid implant to receive FDA approval.^8^ DEXI is effective in improving anatomical and functional outcomes of DME but with associated risks like increased intraocular pressure (IOP) and cataract formation.^9^ Moreover, the duration of action is only about 3-6 months, and deterioration of outcomes is often noted after peak efficacy is reached.^10^

To overcome these drawbacks, the 0.19 mg fluocinolone acetonide implant (FACI) [Iluvien, Alimera Sciences Ltd; Alphargetta GA, USA] has been developed. The kinetics of FACI are slower and peak efficacy is reached at 11 months with slow release of 0.2µg/day of the drug for up to three years.^11^ Guidelines recommend FACI to be used as a third-line of therapy in DME cases.^12^ However, in real-world practice, patients may be shifted to FACI directly after anti-VEGF injections, after DEXI, or even off-label triamcinolone injections.^13^ Reasons for the switch could be treatment resistance, the need for repeated DEXI injections, or steroid challenge before FACI injections. While the FDA has approved the use of FACI in patients previously treated with DEXI without any significant rise in IOP, the modalities of switching from DEXI to FACI are not well described in literature.^14^ It is unclear how patients respond to switching from a short-acting to a long-acting steroid. To fill this gap in the literature, we systematically reviewed published studies and assessed the efficacy and safety of FACI in DME patients previously treated with DEXI.

## METHODOLOGY

We explored the websites of Embase, PubMed, CENTRAL, Web of Science, and Scopus for studies reporting outcomes of FACI in patients previously treated with DEXI. We used a combination of MeSH and free keywords to work out the search strategy which was: (Fluocinolone Acetonide) OR (FACI)) OR (steroid implant)) OR (intravitreal corticosteroid)) OR (drug implant)) AND ((diabetic retinopathy) OR (diabetic macular edema) Two reviewers (WH, LN) replicated this strategy on all databases. The bibliography for potential articles meeting the inclusion criteria was also manually examined. Lastly, a supplemental search was run on Google Scholar for any other potential articles in gray literature. The search was completed on January 15, 2025.

The protocol of the study has been registered in PROSPERO. The registration number was CRD42025635691. The review is reported as per Preferred Reporting Items for Systematic Reviews and Meta-Analyses (PRISMA) guidelines.^15^

The search queries were run on respective databases and all results were collated and deduplicated in EndNote software (version X9.3.3, Thomson Reuters, Philadelphia, USA). The remaining studies were analyzed for eligibility by examining the titles and abstracts. Studies chosen for further analysis by either reviewer were downloaded and full texts were assessed. The final selection was after the agreement of both reviewers. A third reviewer (HY) was included to resolve disagreements.

### Inclusion criteria:

Detailed inclusion criteria were formulated by all reviewers. We included:

- All studies conducted on patients with DME.
- All included patients were previously treated with DEXI and then switched to FACI.
- The studies reported best corrected visual acuity (BCVA) or central retinal thickness (CRT).
- Included a minimum of 10 eyes in the study.

### Exclusion criteria:

- Studies that included patients who were switched to FACI after anti-VEGF injections.
- Studies not mentioning the needed outcomes.
- Studies with duplicate data.
- Studies available as only abstracts and unpublished thesis.

### Data management and risk of bias:

Details of authors, publication date, number of eyes, age, gender, prior treatments (anti-VEGF, vitrectomy, DEXI), DME duration, HbA1c, pseudophakic eyes, use of IOP lowering medication, baseline BCVA and CRT before FAXI treatment, follow-up and outcomes was extracted from the studies. The primary outcome was a change in BCVA and CRT post-FACI vs pre-FACI. Secondary outcomes were IOP and additional treatments like anti-VEGF injections, DEXI, focal laser, and second FACI. We did not make any assumptions about missing data. Studies not reporting data as mean and standard deviation (SD) for BCVA and CRT were omitted from the meta-analysis.

Risk of bias was assessed using the quality assessment tool for before-and-after studies.^16^ Studies were analyzed on following 10 items as shown in Supplementary Table-I.

**Supplementary Table-I:** Risk of bias assessment.

Study	Was the Study Question or Objective Clearly Stated?	Were Eligibility /Selection Criteria for the Study Population Prespecified and Clearly Described?	Were the Participants in the Study Representative of Those Who Would be Eligible for the Intervention in the General or Clinical Population of Interest?	Were all Eligible Participants That Met the Prespecified Entry Criteria Enrolled?	Was the Sample Size Sufficiently Large to Provide Confidence in the Findings?	Was the Intervention Clearly Described and Delivered Consistently Across the Study Population?	Were the Outcome Measures Prespecified, Clearly Defined, Valid, Reliable, and Assessed Consistently Across All Study Participants?	Was the Loss to Follow-up After Baseline 20% or Less?	Did the Statistical Methods Examine Changes in Outcome Measures From Before to After the Intervention? Were Statistical Tests Done That Provided P Values for the Pre-to-Post Changes?	Were Outcome Measures of Interest Taken Multiple Times Before the Intervention and Multiple Times After the Intervention (ie, Did They Use an Interrupted Time-Series Design)?	Rating
Lampin 2025^24^	Yes	Yes	Yes	Yes	No	Yes	Yes	Yes	Yes	Yes	Fair
Rousseau 2023^23^	Yes	Yes	Yes	Yes	Yes	Yes	Yes	Yes	Yes	Yes	Good
Elbarky 2023^22^	Yes	Yes	Yes	Yes	No	Yes	Yes	Yes	Yes	Yes	Fair
Mathis 2022^21^	Yes	Yes	Yes	Yes	Yes	Yes	Yes	Yes	Yes	Yes	Good
Baillif 2022^20^	Yes	Yes	Yes	Yes	Yes	Yes	Yes	Yes	Yes	Yes	Good
Cicinelli 2021^19^	Yes	Yes	Yes	Yes	Yes	Yes	Yes	Yes	Yes	Yes	Good
Vaz-Pereira 2020^18^	Yes	Yes	Yes	Yes	Yes	Yes	Yes	Yes	Yes	Yes	Good
Rehak 2020^17^	Yes	Yes	Yes	Yes	Yes	Yes	Yes	Yes	Yes	Yes	Good

### Statistical analysis:

The software used was “Review Manager” (RevMan, version 5.3) for the primary meta-analysis. We compared post-treatment with pre-treatment data in the meta-analysis for the follow-up intervals of three months, six months, and 12 months. Mean and SD values were entered for BCVA, CRT, and IOP to calculate the difference as Mean Difference (MD) with 95% confidence intervals (CI). Most studies measured BCVA on the Early Treatment Diabetic Retinopathy Study (ETDRS) scale. Therefore, studies reporting data as LogMar were analyzed descriptively. Heterogeneity among studies was assessed through Cochran’s Q statistic and the *I^2^* index. *I^2^* of over 50% and/or *P*<0.05 indicated significant heterogeneity. The influence of individual studies was judged by sensitivity analysis which was done in the Review Manager software itself. One study at a time was removed from the meta-analysis to assess the stability of the results. Due to the small number of studies (<10), the funnel plot was not drawn for publication bias.

## RESULTS

### Database search results:

**Fig.1:**
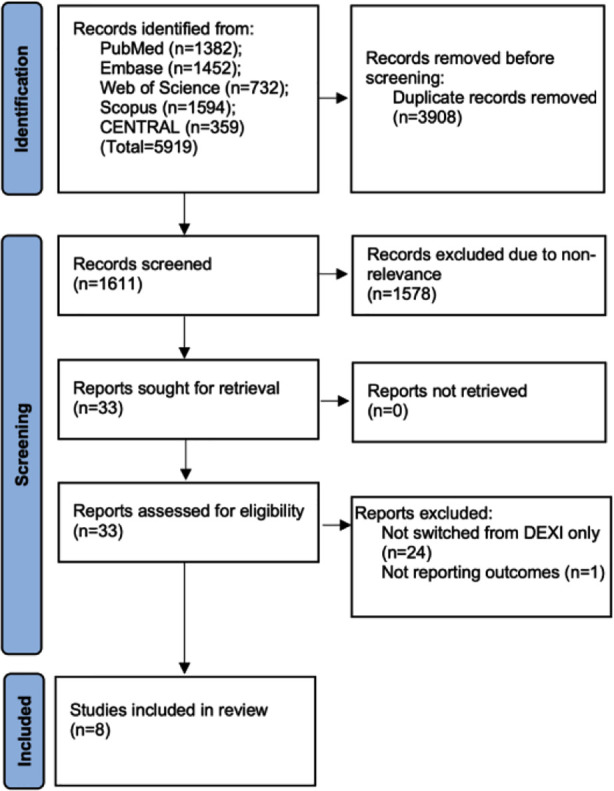
Study flowchart.

As per PRISMA, the flowchart depicting the results at each step of study selection is presented in Fig.1. The reviewers screened 1611 unique articles. Thirty-three of them were found to be relevant to the review and hence were selected for further analysis. In the end, eight studies^17^-^24^ were eligible for the review.

### Baseline details:

Information extracted by the authors from the included studies is shown in Table-I. Most studies were from European countries and only one was from Asia (UAE). One was a prospective study while all others were retrospective. Overall, the studies included 365 eyes. The mean age of patients was > 60 years in all studies. Cumulatively, 52.8% of the population consisted of male patients. Time since DME diagnosis was >39 months in all studies except for Rehak et al.^17^ Diabetes control was good across most studies with baseline HbA1c being <8%. In four studies, all patients had been treated with anti-VEGF, and in another four studies, all had undergone vitrectomy. In three studies, the percentage of patients who had received anti-VEGF was >80%. In total, 88.8% (324/365) of the total eyes had received prior anti-VEGF injections and the same number of eyes had also undergone vitrectomy. Only four studies reported data on the interval between DEXI and FACI. In three studies, the interval was 1-2 months while in another the interval was >4 months. All eyes were pseudophakic in four studies. Overall, 23.2% (85/365) of eyes were on IOP-lowering medications before FACI. Except for one study, all studies had a follow-up of at least 12 months. Most of the studies included were good quality (Supplementary Table-I).

**Table-I:** Details of included studies.

Study	Country	Design	Sample size (eyes)	Mean age	Male (%)	DME duration (m)	HbA1c (%)	Prior anti-VEGF (%)	Prior vitre-ctomy (%)	Mean no of DEXI	Interval DEXI-FACI	Pseudo-phakic (%)	IOP lowering medication (%)	Baseline BCVA (Letters)	Baseline CRT (µm)	F/U (m)
Lampin 2025^24^	France	P	14	67	71	69.5± 4	7.4± 0.9	57	NR	NR	1 m	100	0	65.7± 11.5	358± 130	12
Rousseau 2023^23^	France	R	41	68.7	50	63.8± 22.9	NR	100	NR	6.1 ± 4.5	1 m	100	43.9	63.2 ± 16.6	299.4 ± 103.3	13
Elbarky 2023^22^	UAE	R	22	67	50	NR	NR	100	9	NR	NR	100	0	37.6 ± 15.6	534.3 ± 91.1	24
Mathis 2022^21^	France	R	62	71.6	45.7	60.3± 30.6	7.9± 1.3	83.9	29	NR	NR	96.8	33.9	64.0± 17.2	333.3± 112.5	13.9
Baillif 2022^20^	France	R	113	69.8	48.7	71.8± 48.2	7.4± 0.1	85.8	27.3	6.3± 4.5	1-8 wk	91.2	25.6	54.1± 17.8	454.7± 196.7	12
Cicinelli 2021^19^	Italy	R	44	68.8	59	65.2± 22.2	6.9± 0.8	80	14	4.6± 3.2	>4 m	100	20	0.58 ± 0.44*	478± 151.4	14
Vaz-Pereira 2020^18^	Portugal	R	44	69.2	65.9	39.6± 22.8	NR	100	22.7	1.9± NR	NR	63.6	18.2	41.93± 20.83	542.81± 149.56	8
Rehak 2020^17^	Germany	R	25	62.2	52	6.92± 3.46	7.5± 1	100	0	1.27± 0.14	NR	48	NR	64 ± 9.7	569.6± 127	12

DME, Diabetic macular edema; BCVA, best corrected visual acuity; CRT, central retinal thickness; m, months; wk, weeks; DEXI, dexamethasone implant; FACI, Fluocinolone acetonide implant; VEGF, vascular endothelial growth factor; F/U, follow-up; HbA1c, glycated hemoglobin; R, retrospective cohort; P, prospective cohort, *LogMAR value.

### Outcomes:

**Fig.2:**
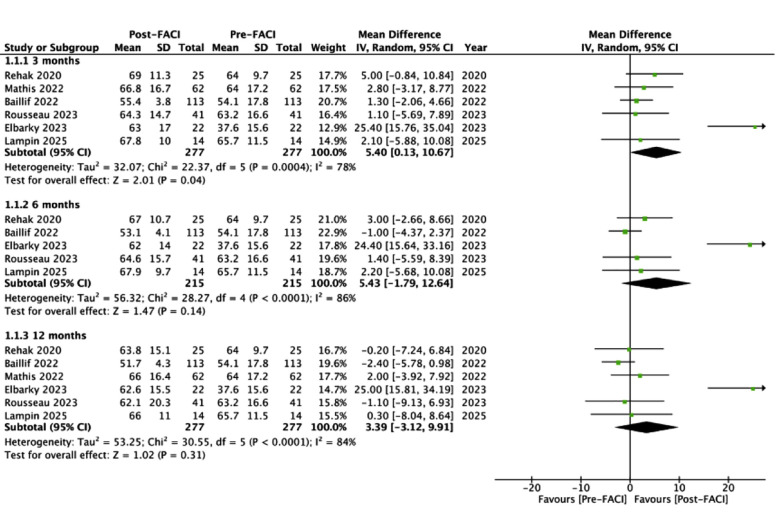
Meta-analysis of change in BCVA (letters) before and after FACI.

Meta-analysis showed that use of FACI was associated with statistically significant improvement in BCVA (letters) at three months (MD: 5.40 95% CI: 0.13, 10.67 I^2^= 78%) but not at six months (MD: 5.43 95% CI: -1.79, 12.64 I^2^=86%), and 12 months (MD: 3.39 95% CI: -3.12, 9.91 I^2^=84%) (Fig.2). The analysis of three months was not robust on sensitivity analysis and turned non-significant on the exclusion of all studies except for Rousseau et al (Data not shown).^23^ The remaining results did not change during sensitivity analysis.

**Fig.3:**
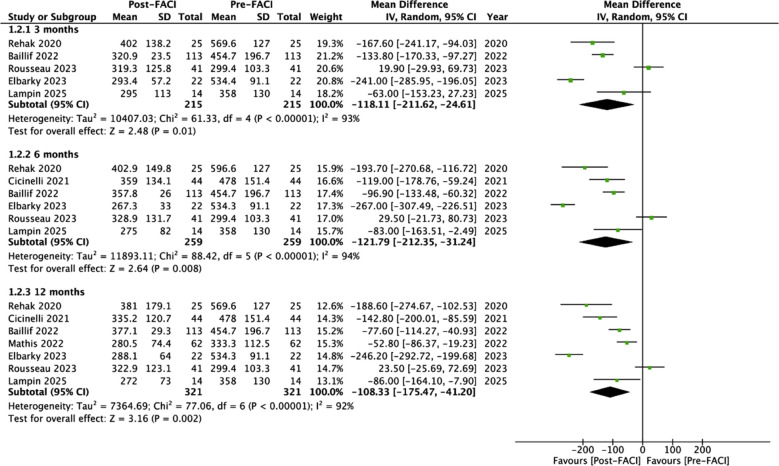
Meta-analysis of change in CRT before and after FACI.

The pooled analysis also indicated a statistically significant reduction in CRT after administration of FACI at 3 months (MD: -118.11 95% CI: -211.62, -24.61 I^2^=93%), six months (MD: -121.79 95% CI: -212.35, -31.24 I^2^=94%), and 12 months (MD: -108.33 95% CI: -175.47, -41.2 I^2^=92%) Fig.3. Herein too, the results of three months were not stable on sensitivity analysis and turned non-significant on exclusion of Rehak et al.,^17^ Bailiff et al.,^20^ and Elbarky A et al.^22^ The results of 6 and 12 months did not change in significance on sensitivity analysis.

The study of Cicinelli et al.^19^ was not included in the meta-analysis of BCVA as it reported data as LogMar. In their study, the authors noted significant improvement in BCVA in the 12 months after shifting to FACI. Another study by Vaz-Pereira et al.^18^ could not be included in the meta-analysis as the authors did not report SD values. The study reported statistically significant improvement in BCVA (mean +6.82 and +13.02 letters; p = 0.005) and CRT (mean -71.81 and -170.77 µm; p = 0.001) at one and six months respectively.

**Fig.4:**
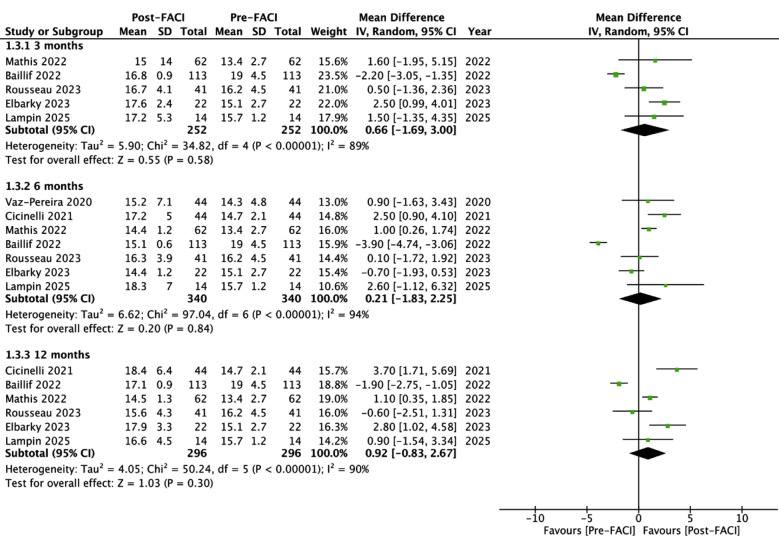
Meta-analysis of change in IOP before and after FACI.

Details of IOP at baseline and follow-up in the included studies are presented in Supplementary Table-II. Meta-analysis showed no statistically significant change in IOP from baseline at three months (MD: 0.66 95% CI: 1.69, 3.00 I^2^=89%), six months (MD: 0.21 95% CI: -1.83, 2.25 I^2^=94%), and 12 months (MD: 0.92 95% CI: -0.83, 2.67 I^2^=90%) Fig-4. All results remained stable on sensitivity analysis. Overall, about 9.7% to 28% of patients had an increase in IOP requiring medication after FACI. Additional treatments were reported by all except for one study (Supplementary Table-II). About 13.7% to 37.1% of patients needed further treatments which included anti-VEGF injections, DEXI, focal laser, and second FACI.

**Supplementary Table-II:** Details of IOP and additional treatments in the included studies.

Study	Baseline IOP	M3 IOP	M6 IOP	M12 IOP	Details of IOP	Additional treatments needed
Lampin 2025^24^	15.7± 1.2	17.2 ± 5.3	18.3 ± 7	16.6 ± 4.5	14% required IOP lowering treatment	21.4%; One eye was treated with aflibercept 2 mg/0.05 mL M5 and M9 and 2 eyes with DEXi at M5 and M11. No patient had retreatment of FACI.
Rousseau 2023^23^	16.2 ± 4.5	16.7 ± 4.1	16.3 ± 3.9	15.6 ± 4.3	Out of 23 eyes not on IOP treatment, 6 (26%) had transient increase in IOP (>21 mmHg) treated with medication.	20.6%; 3 (7.3%) received DEXi injection at M6, 2 (4.9%) received focal laser for TELCAPS at M6 and 1 (2.4%) combined focal laser and DEXI injection at M9.
Elbarky 2023^22^	15.1 ± 2.7	17.6± 2.4	14.4± 1.2	17.9± 3.3	22.7% required IOP lowering treatment	13.7%; all received anti-VEGF
Mathis 2022^21^	13.4± 2.7	15± 1.4	14.4± 1.2	14.5± 1.3	9.7% had an increase in IOP (>21 mmHg) during follow-up and 11.3% had ocular hypertension.	37.1%; 11 (47.8%) received intravitreal aflibercept, 15 (65.2%) received DEX-I, 12 (52.2%) received focal macular laser, and 2 (8.7%) received a second FACI injection.
Baillif 2022^20^	19.0 ± 4.5	16.8± 0.9	15.1± 0.6	17.1± 0.9	29 out of 90 eyes (32.2%) received anti-glaucoma medications. Among them, six were naïve of IOP-lowering drops.	32.7%; macular laser therapy in 3 (8.1%), intravitreal injection of anti-VEGF in 18 (48.6%), and DEXI in 15 (40.5%). One patient was treated with subconjunctival triamcinolone.
Cicinelli 2021^19^	14.7 ± 2.1	NR	17.2 ± 5	18.4 ± 6.4	27% had an IOP > 21 mmHg	25%; all received anti-VEGF
Vaz-Pereira 2020^18^	14.3 ± 4.81	NR	15.2 ± 7.1	NR	25% were managed with IOP lowering drops (three new cases added during follow-up)	NR
Rehak 2020^17^	NR	NR	NR	NR	28% had an increase in IOP (>21 mmHg) during follow-up.	36%; additional DEXI in 8 (32%) and DEXI with two anti-VEGF injections in 1 (4%).

IOP, Intra-ocular pressure; M, month (number ahead of M indicates the month); NR, not reported; DEXI, dexamethasone implant; FACI, Fluocinolone acetonide implant; VEGF, vascular endothelial growth factor.

## DISCUSSION

The efficacy of FACI has been established by several large studies in literature like the FAME trial, USER study, and PALADIN study.^25^-^27^ In most studies, patients were previously treated with either anti-VEGF, DEXI, or off-label triamcinolone injections before FACI.^13^ The USA FDA prescribing guidelines mention that FACI should be used in patients who have been previously treated with a course of corticosteroids and did not have a clinically significant rise in intraocular pressure.^28^ In real-world practice, such corticosteroid exposure may not necessarily be a DEXI implant. Moreover, a large number of clinicians are directly using a FACI after failure with anti-VEGF. One study suggests that prior treatment before FACI was an anti-VEGF agent in 38.9% of eyes and DEXI in 44.4% of eyes.^29^ To date, only one study by Rehak et al.^17^ has compared outcomes of FACI switched from either anti-VEGF or DEXI. While the authors reported similar functional and anatomical outcomes in both groups, data is too scarce for formulating guidelines. In this context, we have presented the first systematic review and meta-analysis of literature examining the efficacy and safety of FACI in patients who were previously treated with DEXI.

The present meta-analysis of single-arm studies included data from 365 eyes from eight studies. A large number of eyes had prior exposure to anti-VEGF (88.8%) before being treated with DEXI, indicating that in most studies, DEXI was the second line of therapy. Importantly, 88.8% of the eyes were also vitrectomized before FACI therapy. However, data on the timing of the switch from DEXI to FACI and the reasons for the same were scarcely reported. Our meta-analysis showed that there was a tendency of BCVA gain after FACI at three, six and 12 months but results were statistically significant only for three months. This outcome was also unstable on sensitivity analysis and turned non-significant on the exclusion of multiple studies. We could observe a peak of 5.4 letter gain with FACI which diminished to a gain of 3.4 letters by 12 months.

The lack of data precluded us from examining outcomes at 24 and 36 months. In comparison, a meta-analysis by Fallico et al.^30^ of real-world FACI studies has shown a mean 4.5 and eight letters gain in BCVA at 24 and 36 months respectively. This was in line with the results of the FAME study which noted a BCVA gain of 4.4 and eight letters respectively.^27^ In the included studies, there was one outlier study of Elbarky A^22^ which reported a large gain (+25 letters) in BCVA at all follow-up intervals. One reason could be the lower baseline BCVA in their study as compared to others causing a greater increase in the first 12 months with a gradual plateau effect on longer follow-up. It is a known fact that eyes with lower baseline BCVA show the largest improvements in BCVA as compared to those with high baseline which show limited gain.^31^

In terms of anatomical response, the meta-analysis showed a statistically significant reduction in CRT at three, six and 12 months after the switch to FACI. Likewise, Fallico et al.^30^ in the meta-analysis of real-world FACI studies have also shown significant reductions in CRT at 24 and 36 months which were more or less similar to the results obtained by the FAME study.^27^ The lack of large change in BCVA and improvements in CRT in our review could be attributed to several reasons. Firstly, the baseline patient characteristics and duration of DME are important factors. Gains in BCVA are strongly correlated with baseline BCVA and are often influenced by the “plateau effect”.^32^ It has also been demonstrated that DME should be promptly treated in the early stages before severe vision loss. A better final BCVA can be expected for short-duration DME.^33^

Secondly, the reason for switching to FACI from DEXI could be for convenience rather than inadequate response. DEXI has greater water solubility as compared to FACI necessitating repeated injections due to loss of efficacy after 3-6 months.^10^ The implant is made up of poly (lactic acid-co-glycolic acid) polymers which disintegrate to water and CO_2_ once the drug is released. A bolus of dexamethasone is delivered after the injection with peak effect at eight weeks.^34^ On the other hand, FACI is made up of a nonbiodegradable polyimide cylinder which is filled by a polymer matrix loaded with the drug. It delivers smaller drug concentrations after the injection (1.26 ng/g) followed by sustained release (0.2µg/day) for 36 months.^11^

Therefore, DEXI is considered more of a bolus treatment while FACI is a basal therapy that stabilizes the disease for a prolonged duration. The longer duration means fewer injections and avoiding repeated ophthalmology consultations. It is plausible that in several patients a switch to FACI may have been for reducing treatment time despite improvements with DEXI. Given the pharmacokinetics of the two drugs, the time interval between DEXI and FACI is also important. In a few studies,^20^,^23^,^24^ FACI was injected within two months of DEXI i.e. during the efficacy period of DEXI without DME recurrence. These patients may have already improved BCVA and CRT and therefore did not demonstrate a major difference in outcomes as compared to those where DME recurred following the decline in the efficacy of DEXI.

Another factor potentially affecting the results is prior vitrectomy which can increase the clearance of intravitreal drugs making intravitreal treatment challenging.^28^ However, a recent comparative study shows that FACI may be equally effective in vitrectomized versus non-vitrectomized eyes with DME. A gain of ≥15 letters was noted in 37.5% of vitrectomized and 36.8% of non-vitrectomized eyes with a CRT change of -217.7± 40.8 µm and -155.6± 43.4 µm respectively.^35^ Another small study by La Mantia et al..^36^ has also shown modest visual and anatomical outcomes with FACI which was not significantly different in vitrectomized versus non-vitrectomized eyes with DME. Due to the high proportion of vitrectomized eyes and lack of separate data, we were unable to perform a subgroup analysis of outcomes.

An increase in IOP and cataracts are the common adverse events of corticosteroid implants. The FAME study noted an IOP increase in 37.1% of subjects receiving FACI with 10% experiencing ≥10 mmHg increase from baseline.^27^ Consistent values have been noted in real-world studies where around 10-15.4% of patients have experienced ≥10 mmHg increase in IOP after FACI.^28^ A prior steroid challenge helps decipher the risk of IOP after FACI as noted in the USER study where fewer patients experienced an increase in IOP.^26^ The PALADIN study also showed that the risk of ocular hypertension is low after FACI if there is no history of the same with prior steroid exposure.^25^ Overall, only 23.2% of participants in the included studies were on IOP-lowering medications before FACI. During the follow-up period, 9.7% to 28% of patients needed IOP-lowering therapy. The wide variation in numbers could be due to the varied real-world characteristics of the patients. However, on pooled analysis, we did not find any statistically significant change in IOP from baseline to up to 12 months after FACI.

### Limitations:

We acknowledge several limitations of the current review. The small number of studies, limited sample size, and retrospective nature of the data preclude strong conclusions. Not all studies reported data for all follow-up intervals and some did not provide complete data which limited inclusion of all eight studies in the meta-analyses. There was obvious selection bias in all included studies as reasons for the switch from DEXI to FACI were not mentioned. The lack of data on the timing of the switch also limits the clarity of outcomes. The included population was also inhomogeneous with variations in DME duration, prior therapies, baseline BCVA and CRT, and follow-up. Given the fact that FACI works for up to three years, analysis of long-term data was necessary to best assess its efficacy. Another important fact is that 13.7% to 37.1% of patients needed further treatments after FACI. Herein too, several individual patient factors play a role like in any real-world setting. Specific reasons for re-treatment were not widely reported and hence could not be further analyzed.

## CONCLUSIONS

The use of FACI after DEXI can lead to significantly improved anatomical but modest gains in functional outcomes in DME patients. The use of FACI does not seem to cause significant change in IOP. Present evidence is from a highly heterogeneous patient population and therefore should be interpreted with caution. Further studies are needed to decipher the most optimal time to switch from DEXI to FACI.

### Authors’ contributions:

**WH:** Study design and manuscript writing, manuscript revision and validation, is responsible for the integrity of the study. **LN, HY, ZZ, ST, YT and LC:** Data collection, data analysis and interpretation. Critical Review.

All authors have read and approved the final manuscript.

## References

[1] Stafford LK, McLaughlin SA, Boyko EJ, Vollset SE, Smith AE, GBD 2021 Diabetes Collaborators KL (2023). Global, regional, and national burden of diabetes from 1990 to 2021, with projections of prevalence to 2050:a systematic analysis for the Global Burden of Disease Study 2021. Lancet (London, England).

[2] Morya AK, Ramesh PV, Nishant P, Kaur K, Gurnani B, Heda A (2024). Diabetic retinopathy:A review on its pathophysiology and novel treatment modalities. World J Methodol.

[3] Chen E, Looman M, Laouri M, Gallagher M, Van Nuys K, Lakdawalla D (2010). Burden of illness of diabetic macular edema:literature review. Curr Med Res Opin.

[4] Romero-Aroca P, Baget-Bernaldiz M, Pareja-Rios A, Lopez-Galvez M, Navarro-Gil R, Verges R (2016). Diabetic Macular Edema Pathophysiology:Vasogenic versus Inflammatory. J Diabetes Res.

[5] Sakini AS Al, Hamid AK, Alkhuzaie ZA, Al-Aish ST, Al-Zubaidi S, Tayem AA (2024). Diabetic macular edema (DME):dissecting pathogenesis, prognostication, diagnostic modalities along with current and futuristic therapeutic insights. Int J Retin Vitr.

[6] Deng J, Qin Y (2024). Advancements and emerging trends in ophthalmic anti-VEGF therapy:a bibliometric analysis. Int Ophthalmol.

[7] Gonzalez VH, Campbell J, Holekamp NM, Kiss S, Loewenstein A, Augustin AJ (2016). Early and Long-Term Responses to Anti-Vascular Endothelial Growth Factor Therapy in Diabetic Macular Edema:Analysis of Protocol I Data. Am J Ophthal.

[8] Spinetta R, Petrillo F, Reibaldi M, Tortori A, Mazzoni M, Metrangolo C (2023). Intravitreal DEX Implant for the Treatment of Diabetic Macular Edema:A Review of National Consensus. Pharmaceutics.

[9] Yan A, Jones C, Demirel S, Chhablani J Diabetic macular edema:Upcoming therapies. Graefe's Arch Clin Exp Ophthalmol.

[10] Bastakis GG, Dimopoulos D, Stavrakakis A, Pappas G (2019). Long-term efficacy and duration of action of dexamethasone implant, in vitrectomised and non-vitrectomised eyes with persistent diabetic macular oedema. Eye (London, England).

[11] Campochiaro PA, Brown DM, Pearson A, Chen S, Boyer D, Ruiz-Moreno J (2012). Sustained delivery fluocinolone acetonide vitreous inserts provide benefit for at least 3 years in patients with diabetic macular edema. Ophthalmol.

[12] Cicinelli MV, Rabiolo A, Capone L, Di Biase C, Lattanzio R, Bandello F (2023). Factors associated with the response to fluocinolone acetonide 0.19mg in diabetic macular oedema evaluated as the area-under-the-curve. Eye (Lond).

[13] Taloni A, Coco G, Rastelli D, Buffon G, Scorcia V, Giannaccare G (2023). Safety and Efficacy of Dexamethasone Intravitreal Implant Given Either First-Line or Second-Line in Diabetic Macular Edema. Patient Prefer Adherence.

[14] Kodjikian L, Bandello F, de Smet M, Dot C, Zarranz-Ventura J, Loewenstein A (2022). Fluocinolone acetonide implant in diabetic macular edema:International experts'panel consensus guidelines and treatment algorithm. Eur J Ophthalmol.

[15] Page MJ, McKenzie JE, Bossuyt PM, Boutron I, Hoffmann TC, Mulrow CD (2021). The PRISMA 2020 statement:An updated guideline for reporting systematic reviews. Inter J Surg.

[16] National Heart Lung and Brain Institute Study Quality Assessment Tools.

[17] Rehak M, Busch C, Unterlauft JD, Jochmann C, Wiedemann P (2020). Outcomes in diabetic macular edema switched directly or after a dexamethasone implant to a fluocinolone acetonide intravitreal implant following anti-VEGF treatment. Acta Diabetol.

[18] Vaz-Pereira S, Castro-De-Sousa JP, Martins D, Prates Canelas J, Reis P, Sampaio A (2020). The Outcomes of Switching from Short- to Long-Term Intravitreal Corticosteroid Implant Therapy in Patients with Diabetic Macular Edema. Ophthal Res.

[19] Cicinelli MV, Rosenblatt A, Grosso D, Zollet P, Capone L, Rabiolo A (2021). The outcome of fluocinolone acetonide intravitreal implant is predicted by the response to dexamethasone implant in diabetic macular oedema. Eye (Basingstoke).

[20] Baillif S, Staccini P, Weber M, Delyfer MN, Le Mer Y, Gualino V (2022). Management of Patients with Diabetic Macular Edema Switched from Dexamethasone Intravitreal Implant to Fluocinolone Acetonide Intravitreal Implant. Pharmaceutics.

[21] Mathis T, Papegaey M, Ricard C, Rezkallah A, Matonti F, Sudhalkar A (2022). Efficacy and Safety of Intravitreal Fluocinolone Acetonide Implant for Chronic Diabetic Macular Edema Previously Treated in Real-Life Practice:The REALFAc Study. Pharmaceutics.

[22] Elbarky A (2023). Effectiveness and tolerability of the fluocinolone acetonide implant in patients with diabetic macular edema in the UAE:24 and 36-month outcomes. Eur J Ophthalmol.

[23] Rousseau N, Lebreton O, Masse H, Maucourant Y, Pipelart V, Clement M (2023). Fluocinolone Acetonide Implant Injected 1 Month after Dexamethasone Implant for Diabetic Macular Oedema:the ILUVI1MOIS Study. Ophthalmol Ther.

[24] Lampin Q, Poret J, Gherras M, Jany B, Tran THC (2025). Fluocinolone acetonide implant (FAci) one month after dexamethasone implant (DEXi) for chronic diabetic macular edema:1-year results. J Fr d'Ophtalmol.

[25] Mansour SE, Kiernan DF, Roth DB, Eichenbaum D, Holekamp NM, Kaba S (2021). Two-year interim safety results of the 0.2 µg/day fluocinolone acetonide intravitreal implant for the treatment of diabetic macular oedema:the observational PALADIN study. Br J Ophthalmol.

[26] Eaton A, Koh SS, Jimenez J, Riemann CD (2019). The USER Study:A Chart Review of Patients Receiving a 0.2 µg/day Fluocinolone Acetonide Implant for Diabetic Macular Edema. Ophthalmol Ther.

[27] Campochiaro PA, Brown DM, Pearson A, Ciulla T, Boyer D, Holz FG (2011). Long-term benefit of sustained-delivery fluocinolone acetonide vitreous inserts for diabetic macular edema. Ophthalmol.

[28] Syed YY (2017). Fluocinolone Acetonide Intravitreal Implant 0.19 mg (ILUVIEN®):A Review in Diabetic Macular Edema. Drugs.

[29] McCluskey JD, Kaufman PL, Wynne K, Lewis G (2019). Early adoption of the fluocinolone acetonide (FAc) intravitreal implant in patients with persistent or recurrent diabetic macular edema (DME). Inter Med Case Rep J.

[30] Fallico M, Maugeri A, Lotery A, Longo A, Bonfiglio V, Russo A (2021). Fluocinolone acetonide vitreous insert for chronic diabetic macular oedema:a systematic review with meta-analysis of real-world experience. Sci Rep.

[31] Alfaqawi F, Lip PL, Elsherbiny S, Chavan R, Mitra A, Mushtaq B (2017). Report of 12-months efficacy and safety of intravitreal fluocinolone acetonide implant for the treatment of chronic diabetic macular oedema:a real-world result in the United Kingdom. Eye (London, England).

[32] Dugel PU, Hillenkamp J, Sivaprasad S, Vögeler J, Mousseau MC, Wenzel A (2016). Clinical Ophthalmology Dovepress Baseline visual acuity strongly predicts visual acuity gain in patients with diabetic macular edema following anti-vascular endothelial growth factor treatment across trials. Clin Ophthalmol.

[33] Kodjikian L, Baillif S, Creuzot-Garcher C, Delyfer MN, Matonti F, Weber M (2021). Real-World Efficacy and Safety of Fluocinolone Acetonide Implant for Diabetic Macular Edema:A Systematic Review. Pharmaceutics.

[34] Whitcup SM, Cidlowski JA, Csaky KG, Ambati J (2018). Pharmacology of Corticosteroids for Diabetic Macular Edema. Invest Ophthalmol Vis Sci.

[35] Pessoa B, Coelho J, Correia N, Ferreira N, Beirão M, Meireles A (2018). Fluocinolone Acetonide Intravitreal Implant 190 μg (ILUVIEN®) in Vitrectomized versus Nonvitrectomized Eyes for the Treatment of Chronic Diabetic Macular Edema. Ophthalmic Res.

[36] La Mantia A, Hawrami A, Laviers H, Patra S, Zambarakji H (2018). Treatment of refractory diabetic macular edema with a fluocinolone acetonide implant in vitrectomized and non-vitrectomized eyes. Interl J Ophthalmol.

